# **Interdisciplinarity in cultural gerontology and geriatric medical humanities** –** a bibliometric survey**

**DOI:** 10.1007/s41999-024-01053-3

**Published:** 2024-09-19

**Authors:** Emily Waldron, Katherine Wakefield, Desmond O’Neill

**Affiliations:** Centre for Ageing, Neuroscience and the Humanities, Trinity College Dublin, Trinity Centre for Health Science, Tallaght University Hospital, Dublin, D24 NR0A Ireland

**Keywords:** Aging, Humanities, Interdisciplinary studies

## Abstract

**Background:**

Cultural gerontology – understanding ageing through the lens of arts and humanities—has emerged as an important element of epistemology of ageing. As a boundary area between geriatric medicine/gerontology and arts/humanities disciplines, joint-working/interdisciplinarity is desirable. This project aims to assess the degree of joint-working manifested in cultural gerontology by authorship and acknowledgements in papers dedicated to cultural gerontology in five journals.

**Methods:**

Observational survey of authorship in 5 journals from the founding of the specific sections on cultural gerontology or specific dedicated journals, assessing number of authors, disciplinary identities, and evidence of joint working within cultural gerontology.

**Results:**

Of 591 papers, 481 (81%) were single authors. There was a spread of disciplinary affiliations, 247 (41.8%) gerontology/age studies, 169 arts/humanities/social sciences (28.6%) and 133 of uncertain affiliation (22.5%): only 38 papers had a clear indication of joint working across the disciplines (6.4%). In the two geriatric medicine journals, European Geriatric Medicine and Journal of the American Geriatrics Society, authorship was almost exclusively from geriatric medicine/gerontology. There was extremely limited use of acknowledgements.

**Conclusion:**

Our study indicates that single authorship is the most frequent mode of peer-reviewed publishing in cultural gerontology, whilst acknowledging that some authors may have scholarly training in multiple fields but are listed as unidisciplinary. Leaders in the field and editors of relevant journals/section need to consider ways of encouraging and recognising joint working, through fuller descriptions of multiple affiliations, brief author biographies, fuller use of acknowledgements and consideration of brief accompanying discussant responses from complementary disciplines.

## Background

Cultural gerontology – understanding ageing through the lens of arts and humanities—has emerged as an important element of epistemology of ageing [1]. As a boundary area between geriatric medicine/gerontology and arts/humanities disciplines, joint working/interdisciplinarity is desirable. A general challenge in the broader field of medical humanities is a lack of joint working between healthcare disciplines and scholars in the arts and humanities [2, 3]*.* This survey aims to assess the degree of joint working manifested in cultural gerontology by authorship and acknowledgements in papers dedicated to cultural gerontology in five journals. Although cultural gerontology occurs episodically in other gerontology and generalist journals, we considered that the specificity of focus on cultural gerontology in the five journals provided the most important focus of study.

Although, there has been an acknowledgement of the necessity of interdisciplinarity within ageing research [4], this bibliometric survey aims to determine whether similar developments have been made in cultural gerontology. Within other medical areas, such as psychiatry, it has been shown that collaborative work with the arts and humanities is beneficial [5], and the implications of this in cultural gerontology should be further studied.

## Methods

Observational survey of authorship in 5 journals either dedicated to cultural gerontology or with a dedicated section for cultural gerontology: ‘European Geriatric Medicine (EGM),’ ‘Age, Culture, Humanities: An Interdisciplinary Journal (ACH),’ ‘Journal of the American Geriatric Society (JAGS),’ Journal of Aging, Humanities and the Arts (JAHA),’ and ‘The Gerontologist (TG).’ Articles were included from the founding of the specific sections on cultural gerontology or specific dedicated journals, assessing number of authors, disciplinary identities, and evidence of joint working within cultural gerontology. EGM is affiliated with the European Geriatric Medicine Society; it’s focus is primarily geriatric medicine rather than gerontological. Age, Culture, Humanities is a Danish journal with both European and North American affiliations that publishes articles on older age from an arts and humanities perspective, with a focus on interdisciplinarity. The Journal of the American Geriatrics Society is a North American-based journal that publishes clinical research concerning older people and the ageing process; the journal is aimed at medical professionals and covers advances in the field of geriatric medicine. The Journal of Aging, Humanities, and the Arts was a North America-based journal published by the Humanities and Arts Committee of the Gerontological Society of America from 2007–2010. The journal focussed on inter-disciplinary research into age and ageing between sciences and humanities. The Gerontologist is also affiliated with The Gerontological Society of America: its focus is on social issues related to older people and ageing, with a multidisciplinary focus.

For JAGS we included all relevant articles within the ‘Ars Longa: Humanities and Aging’ section from the founding of the rubric in 2021, all relevant articles from ACH from its founding in 2014, all articles within the section ‘Geriatric Medical Classics’ in EGM from the founding of the rubric in 2019, and all humanities related articles in TG from 2011 until March 2023. We extracted the following variables from each published paper: number of authors (single or multiple); type of article (book review, film review, original article, research article, essays), proportion of each article type within a journal, author affiliations; number of disciplines listed per author. We grouped authors declared as gerontology, age studies, geriatrics, and health and social science disciplines together, and those declared as humanities and arts together.

We also surveyed the degree to which collaboration, if any, was attributed in the acknowledgements of the papers we studied. We did this because acknowledgements often contain useful additional information which can be a method of identifying joint working [6]. If there was any discrepancy/uncertainty about any classifications of affiliations/acknowledgments or determining if an article was to be included, it was discussed by the two primary authors with a final adjudication from the senior author (DON). Data were recorded and analysed using Microsoft Excel software. As this was a desk-based research bibliometric survey, ethical approval was not required.

## Results

Of 591 papers, 481 (81%) declared a single author. There was a spread of disciplinary affiliations, 247 (41.8%) gerontology/age studies, 169 arts/humanities/social sciences (28.6%) and 133 of uncertain affiliation (22.5%): only 38 papers had a clear indication of joint working across the disciplines (6.4%). In the two geriatric medicine journals, EGM and JAGS, authorship was almost exclusively from geriatric medicine/gerontology. There was extremely limited use of acknowledgements recognising complementary disciplines.

In ACH there was a total of 66 single author papers meeting our requirements, of which 46 had authors affiliated with the humanities, 18 with gerontology/age studies, and two of unspecified discipline. Of 11 papers written by multiple authors, three had authors only affiliated with humanities, whilst none were written by authors only affiliated as gerontology/age studies. Seven studies were written by a combination of age studies/gerontology and humanities affiliated authors, while one was authored by a combination of age studies/gerontology affiliated authors and authors of unspecified affiliation. This came to a total of 77 papers from this journal, of which 42 were book reviews (54.5% of papers). The range of authors was 1–3, whilst the average number of authors on multiple author papers was 2.14. There were no disciplinary acknowledgements recorded.

Of 140 papers in JAHA, 110 were single author papers and 30 had multiple authors. Of single author papers, 38 were by humanities affiliated authors, 28 were by gerontology/age studies affiliated authors, and 44 unspecified. The numbers of ranged from 1–6, with an average of 2.93 authors in multiple author papers. Of the multiple author papers seven were by gerontology/age studies affiliated authors, five were by humanities affiliated authors, and eight were written by a combination of humanities and gerontology/age studies affiliated authors: affiliation was not specified in ten of the multiple author papers. There were 14 book reviews, of which 13 were written by a single author, and 1 by multiple authors. Of three film reviews, one declared multiple authors and two single authors. Overall the 17 reviews accounted for 12.1% of papers. There were no clear disciplinary affiliations in acknowledgements.

Of 11 papers in EGM, four were single author and seven had multiple authors, with a range of 1–3. All papers were written by authors with healthcare affiliations and no disciplinary acknowledgements were recorded.

There were 278 single author papers and 55 multiple author papers included from TG. Of single author papers, 127 were book, and 110 film, reviews: there were only two double author book reviews and 1 book review with three authors. Overall, 237 reviews constituted 72.1% of relevant papers, 85.3% of single author papers and 5.5% of multi-author papers. Of the single author papers, 63 were by humanities affiliated authors, 142 by gerontology/ageing studies affiliated authors, and 3 by authors with both humanities and gerontology/ageing studies affiliations. No disciplinary affiliation was noted for 70 single author papers. Of the multiple author papers, 17 were by gerontology/age studies affiliated authors only, 12 were by humanities affiliated authors only, and 19 were by a combination of humanities and gerontology/age studies affiliated authors, with the remaining seven by authors of uncertain discipline. There were three age studies/gerontology acknowledgements, and one arts and humanities acknowledgement in this journal.

Of 30 papers from JAGS, 23 were single author and seven had multiple authors, with a range of 1–7. Of single author papers, 22 authors had healthcare affiliations and one a humanities affiliation. Of the multiple author papers only one paper had both humanities and healthcare affiliated authors, and the average number of authors on a multi-author paper was 2.86. There was only one acknowledgement of note recorded which was to a humanities affiliated contributor. Of the papers included from this journal there were two book reviews and two film reviews; the book reviews were both single author, whilst one film review was single author and one had multiple authors.

## Discussion

Interdisciplinarity appears to be relatively rare in the literature of cultural gerontology. There are likely several reasons for this lack of interdisciplinary engagement in authorship.

One factor is that in some fields within humanities disciplines, appointment and promotion decisions may favour single authorship [7, 8]. This pressure may lead to authors avoiding recognition of interdisciplinary working to meet promotion and reappointment criteria. We suggest that in this complicated era of scholarship, in order to promote interdisciplinarity, editorial leadership of peer-reviewed journals should encourage transparency in authorship [9] and promote support from other disciplines (more value should be placed on joint authorship papers) [10]. Possible solutions to increase interdisciplinarity within authorship and enhance collaborations between the humanities, clinicians, and gerontologists, and could include holding educational seminars/conferences to promote joint working and interdisciplinary relationships. Examples specific to cultural gerontology include the meetings of the European (https://temp.agingstudies.eu/) and North American Networks on Aging (https://agingstudies.org/NANAS/), as well as learning from broader medical humanities meetings. There has been a specific focus on cultural gerontology in the Gerontological Society of America for over five decades [11] and signs of emerging interest in some geriatric medicine societies for formal special interest groups.

Hopefully these conferences would encourage the exchange of knowledge between different institutes/disciplines and promote joint publishing. Also, further research should examine the barriers that prevent joint working [12] to allow these barriers to be removed as well as establishing facilitators to joint working.

More papers could use the approach of ACH which encourages short biographies of the authors with information on scholarly experience and affiliations. There could also be the use of detailed descriptions of the roles that each of the authors had in producing the paper, so that it is clear whether the joint work constitutes an interdisciplinary (involving connected contributions from different disciplines) or multidisciplinary effort [13]. In addition to this a clearer definition of and distinction between the different types of joint working eg., interdisciplinarity, multidisciplinary, would be welcomed. We also suggest more frequent and detailed use of acknowledgements. There was very limited acknowledgement of disciplinary contributions in any of the journals- across all journals, except EGM, single authorship was the most prevalent paper type. Even though the majority of papers in the journal of EGM had multiple authors, there was no arts/humanities authorship in any of the articles, and thus no obvious evidence of joint working in the papers. We recognise that there will always be a role and need for single authorship papers; however, we believe it is likely that more collaborative styles will increase the quality and range of papers published within cultural gerontology and geriatric medical humanities.

A helpful evolution in promoting interdisciplinarity has been a series of iterations of the key North American text in humanities and ageing. Starting with creating a strong argument for the legitimacy of a humanities approach to ageing in the first edition, the second edition included a stronger emphasis on literary studies and imaginative sociocultural analysis, heralding the nexus of ‘critical’ turn in age studies highlighting the opportunities offered by a humanities approach. However, both this and the subsequent editions did not quite bridge the gap with the broader field of gerontology. The fourth edition has taken a more structured interdisciplinary approach, including commentaries from scholars in complementary fields for each chapter to develop a cross-disciplinary view[14]*.*

Our study had a number of strengths, such as the large number of papers (591) included that were published over a period of 12 years. The broad categorisation we applied also allowed us to follow general trends in authorship in a way that wouldn't be possible without categorising the disciplines.

As well as this, our examination of disciplinary acknowledgements in addition to authorship was a strength as it provided us another way of recognising contributors to the papers.

Some limitations of our study include the fact that we only included articles from 5 journals, and as we focussed on authorship since 2011 we may have missed earlier trends in joint working within gerontology prior to this. Our methodology may not have identified all interdisciplinarity as we did an analysis of self-declared affiliations/disciplines rather than a textual analysis of papers for evidence of joint-working in the content rather than in the author list or acknowledgements.

Different journals had different details of affiliation/discipline therefore our categorisation and recognition of interdisciplinarity was limited by this. In addition to this, not all listed affiliations were clearly associated with an academic discipline, and thus were recorded as unspecified.

By categorising the authors’ different affiliations under the broad headings ‘Arts/Humanities,’ ‘Gerontology/Age Studies,’ and ‘Unspecified,’ we may have missed cross-disciplinary authorship within these categories.

## Conclusion

Our study indicates that single authorship is the most frequent mode of peer-reviewed publishing in cultural gerontology, whilst acknowledging that some authors may have scholarly training in multiple fields but are listed as unidisciplinary. There will always be a role and need for single authorship papers however we believe it is likely that more collaborative styles will increase the quality and range of papers published within gerontology. Leaders in the field and editors of relevant journals/sections need to consider ways of encouraging and recognising joint working, through fuller descriptions of multiple affiliations, brief author biographies, fuller use of acknowledgements and consideration of brief accompanying discussant responses from complementary disciplines.
